# Broadened quantum critical ground state in a disordered superconducting thin film

**DOI:** 10.1038/s41467-024-46628-7

**Published:** 2024-03-16

**Authors:** Koichiro Ienaga, Yutaka Tamoto, Masahiro Yoda, Yuki Yoshimura, Takahiro Ishigami, Satoshi Okuma

**Affiliations:** Department of Physics, Tokyo Institute of Technology, 2-12-1 Ohokayama, Meguro-ku, Tokyo 152-8551 Japan

**Keywords:** Superconducting properties and materials, Phase transitions and critical phenomena, Two-dimensional materials

## Abstract

A superconductor-insulator transition (SIT) in two dimensions is a prototypical quantum phase transition (QPT) with a clear quantum critical point (QCP) at zero temperature (*T* = 0). The SIT is induced by a field *B* and observed in disordered thin films. In some of weakly disordered or crystalline thin films, however, an anomalous metallic (AM) ground state emerges over a wide *B* range between the superconducting and insulating phases. It remains a fundamental open question how the QPT picture of the SIT is modified when the AM state appears. Here we present measurements of the Nernst effect *N*, which has great sensitivity to the fluctuations of the superconducting order parameter. From a thorough contour map of *N* in the *B*-*T* plane, we found a thermal-to-quantum crossover line of the superconducting fluctuations, a so-called ghost-temperature line associated with the QPT, as well as a ghost-field line associated with a thermal transition. The QCP is identified as a *T* = 0 intercept of the ghost-temperature line inside the AM state, which verifies that the AM state is a broadened critical state of the SIT.

## Abstract (web summary)

The authors present Nernst measurements on a 2D film of amorphous Mo_x_Ge_1−x_, which shows a magnetic-field-induced superconductor-metal-insulator transition. The intermediate metal phase is known as the “anomalous metal” (AM) state. The authors conclude that the AM state originates from broadening of the superconductor-insulator transition.

## Introduction

A quantum phase transition (QPT) between competing ground states at zero temperature (*T* = 0) is a central paradigm seen in a variety of systems such as magnetic materials, cold atom gases, and two dimensional (2D) electrons^1–3^. A prototypical example is a field(*B*)-induced superconductor-insulator transition (SIT) observed in disordered 2D superconductors, which shows a clear quantum critical point (QCP) of (2 + 1)D quantum systems^4–6^. Since the localization theory prohibits a metallic ground state in 2D systems^7^, quantum fluctuations drive a direct transition between condensation and localization of electrons, leading to metallic dissipation with a saturated resistance only at the QCP.

However, an anomalous metallic (AM) ground state emerges unexpectedly over a wide *B* range in weakly disordered amorphous films^8–11^, crystalline 2D superconductors^12–14^, and Josephson junction arrays (JJAs)^15–17^. The origin of the superconductor-metal-insulator transition (SMIT) has been debated^8–22^ mainly from the following two viewpoints. One is a mechanism of the metallic dissipation at *T* = 0. Some of theories predicted that it is attributed to quantum creep of vortices^18–20^, and recent experiments have detected its convincing evidence^10,15,16^. The more fundamental viewpoint is how the QPT picture is modified in the SMIT. A plausible explanation is that the AM state originates from broadening of the SIT^10,19,20^. An existence of such a broadened critical ground state, a so-called quantum critical phase, has attracted much attention in heavy fermion compounds and organic magnets^23,24^. To verify this scenario, the QCP must be found inside the broadened critical state. However, clear experimental evidence is still lacking because the critical scaling analysis for resistance data that is frequently used to determine the QCP of the SIT fails for the SMIT^9^. Furthermore, there are other possibilities including a two-step transition with two QCPs^25^, and a single QCP at the metal-insulator transition point^17^. Therefore, it is indispensable to detect the exact location of the QCP using techniques applicable to 2D films beyond the resistance measurements.

A direct way to detect the QCP is to reveal the quantum critical fluctuations of an order parameter around the QCP. A correlation length of the quantum fluctuations diverges at the QCP as a function of a non-thermal parameter such as *B*^1–3^, just like a correlation length of the thermal fluctuations diverges at a thermal transition temperature as a function of *T*. Two types of fluctuations exist in the superconducting order parameter $\Psi=| \Psi | \exp (i\theta )$. The fluctuations of the amplitude ∣Ψ∣ arise from short-lived Cooper pairs that persist deep inside the normal state, and the fluctuations of the phase *θ* originate from mobile vortices in a vortex-liquid state. In previous reports for 3D superconductors, the thermal critical behavior of the amplitude fluctuations was detected clearly through the Nernst effect above a mean-field transition temperature *T*_c0_, which leads to an exact determination of *T*_c0_^26–33^. These studies utilized a high sensitivity of the Nernst effect to the fluctuations. The Nernst signals generated by the superconducting fluctuations are much larger than the signals from normal electrons as reported in many experimental^10,24,26–38^ and theoretical works^39–45^. This is in contrast to the resistivity and magnetic susceptibility, where a weak contribution of the fluctuations is buried in the strong intensity from normal electron scattering and the Pauli susceptibility, respectively.

In this work, we have applied the measurements of the Nernst effect to a 2D film of amorphous (a-)Mo_*x*_Ge_1−*x*_ with a thickness of 10 nm, which shows the *B*-induced SMIT^8–10^, down to 0.1 K in the quantum regime. By revealing an evolution of the quantum fluctuations across the SMIT, we have found that the QCP is located inside the AM state. The result verifies the view that the AM state is caused by a broadening of the SIT. This work also shows that the Nernst effect is a very useful method to detect a QCP in superconducting systems as reported for the SIT^38^ as well as in strongly correlated systems^37,46^.

## Results

**Fig. 1:**
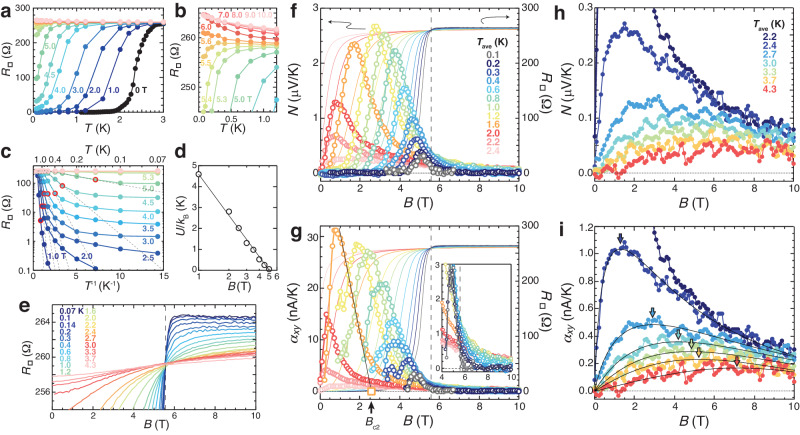
Resistance and Nernst effect in a 2D superconductor exhibiting the field-induced SMIT. **a** The *T* dependence of *R*_□_ in different *B*. *R*_□_ in *B* = 0 was taken as a function of *T* and *R*_□_ in *B* ≠ 0 are plotted from MR in (**e**) and Supplementary Fig. S1. **b** The high-*B* region of (**a**). **c** An Arrhenius plot of (**a**). Open red circles denote *T*_cross_ at which *R*_□_ deviates from a thermally activated form drawn by dashed lines. **d** The *B* dependence of *U* in the thermally activated form. A black line shows a theoretical fit^8,47^. **e** An enlarged view of MR curves at different *T*. All the MR curves cross at *B*_MI_ indicated by a dashed line. *N* (**f**) and *α*_*xy*_( = *N*/*R*_□_) (**g**) as a function of *B* at different *T* (≤2.4 K). The MR curve at each *T* is also shown, where the lines with different colors correspond to the data of *N* and *α*_*xy*_ with the same colors. A black straight line in (**g**) shows an example for a linear extrapolation of *α*_*xy*_ to determine *B*_c2_. In the inset of (**g**), *α*_*xy*_ in the high-*B* region is enlarged. Vertical dashed lines indicate *B*_MI_. The *B* dependence of *N* (**h**) and *α*_*xy*_ (**i**) at high temperatures (*T* ≥ 2.2 K). Black curves in (**i**) are drawn by an ad hoc function to extract B^*^ as a peak field as indicated by colored arrows (see main text).

**Fig. 2:**
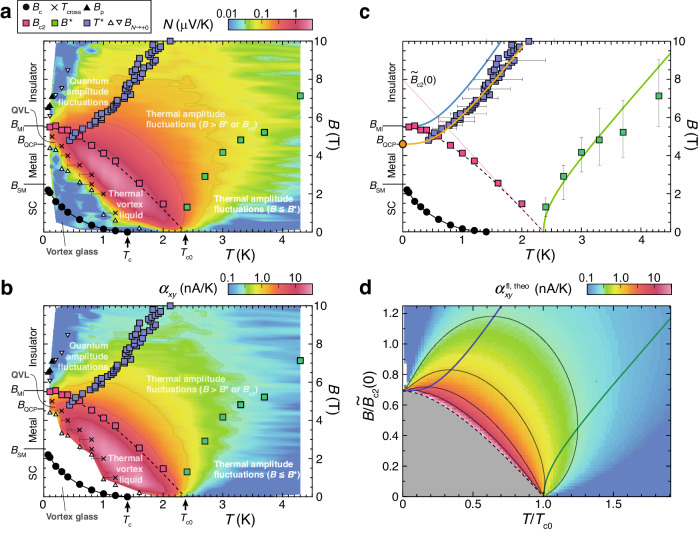
Superconducting fluctuations revealed from the Nernst effect. Contour maps of *N* (**a**) and *α*_*xy*_ (**b**) constructed from Fig. 1f−i. Black circles, crosses, and black triangles denote *B*_c_, *T*_cross_, and *B*_p_ determined from *R*_□_, respectively. White triangles and white inverted triangles represent *B*_*N*→+0_ obtained from *N*. Red, green, and blue squares show *B*_c2_, B^*^, and T^*^, respectively, which are determined from *α*_*xy*_. A dashed curve is a fit by WHH theory^52^. **c** The characteristic temperatures and fields obtained experimentally and plotted in (**a**, **b**) are extracted and displayed in the *B*-*T* plane. Also, theoretically obtained ${B}_{{{{{{{{\rm{theo}}}}}}}}}^{*}$ and ${T}_{{{{{{{{\rm{theo}}}}}}}}}^{*}$ extracted from (**d**) are plotted as green and blue lines, respectively. A dotted red line is an extrapolation of the linear GL region of *B*_c2_(*T*). An orange line is a fit to T^*^ by vertically shrinking the ${T}_{{{{{{{{\rm{theo}}}}}}}}}^{*}$ line ($\equiv {T}_{{{{{{{{\rm{theo}}}}}}}},{{{{{{{\rm{m}}}}}}}}}^{*}$). A solid orange circle indicates *B*_QCP_. Error bars for B^*^ and T^*^ represent ranges of *B* and *T* in which *α*_*xy*_(*B*) at fixed *T* and *α*_*xy*_(*T*) at fixed *B* exceed 95 % of their peak amplitudes, respectively. **d** A theoretical result of ${\alpha }_{xy}^{{{{{{{{\rm{fl}}}}}}}}}(T,B)(\equiv {\alpha }_{xy}^{{{{{{{{\rm{fl}}}}}}}},{{{{{{{\rm{theo}}}}}}}}}(T,B))$ calculated for a 2D superconductor based on the Gaussian fluctuations^43,44^ using the codes provided in ref. ^43^. Note that the input parameters are only *T*_c0_ = 2.36 K and *B*_c2_(0) = 5.5 T obtained experimentally. The *T* and *B* axes are normalized by *T*_c0_ and ${\widetilde{B}}_{{{{{{{{\rm{c2}}}}}}}}}(0)$, respectively. Green and blue lines represent the theoretically obtained ${B}^{*}(\equiv {B}_{{{{{{{{\rm{theo}}}}}}}}}^{*})$ and ${T}^{*}(\equiv {T}_{{{{{{{{\rm{theo}}}}}}}}}^{*})$ respectively. A blank area near *T* = 0 indicates negative *α*_*xy*_ due to strong quantum fluctuations^44^.

Figure 1a, b show the *T*-dependent sheet resistance *R*_□_(*T*) in different *B*. *R*_□_(*T*) in *B* = 0 was measured as a function of *T*, while *R*_□_(*T*) in *B* ≠ 0 was converted from the magnetoresistance (MR) in Fig. 1e and Supplementary Fig. S1. A mean-field transition temperature *T*_c0_( ≡ *T*_c0,*R*_) and a zero-resistance temperature *T*_c_ in *B* = 0 are 2.36 and 1.40 K, respectively (see Methods). With increasing *B*, the transition curve shifts to the low-*T* region. We plot *T*_c_(*B*)( ≡ *B*_c_(*T*)) in a *B*-*T* plane (Fig. 2a–c) with solid black circles (see Supplementary Fig. S1). *B*_c_(*T*) corresponds to a boundary between the vortex-glass phase with zero resistance and the vortex-liquid phase with nonzero resistance. By linearly extrapolating *B*_c_(*T*) to *T* = 0, *B*_c_(0) is estimated to be 2.5 T.

Above *B*_c_(0), *R*_□_(*T* → 0) saturates to nonzero resistance lower than *R*_□_(3.0 K) ( ≡ *R*_□,n_) as clearly seen in an Arrhenius plot (Fig. 1c), indicating that *B*_c_(0)( ≡ *B*_SM_) is a boundary field separating the superconducting phase and the AM state. Dashed straight lines in Fig. 1c represent a thermally activated form ${R}_{\square }={R}_{\square }^{{\prime} }\exp (-U/{k}_{{{{{{{{\rm{B}}}}}}}}}T)$, where ${R}_{\square }^{{\prime} }$ is a coefficient, *U* an activation energy for vortex motion, and *k*_B_ the Boltzmann constant. Deviations from the dashed straight lines are marked with open red circles, which correspond to crossover temperatures *T*_cross_ from the thermal to the quantum creep regime. They are plotted with crosses in the *B*-*T* plane in Fig. 2a, b. *U* extracted from Fig. 1c is plotted as a function of *B* in Fig. 1d. The data points are well fitted by $U={U}_{0}\ln ({B}_{0}/B)$^8,47^ with *U*_0_/*k*_B_ = 2.9 K and *B*_0_ = 5.0 T as indicated by a straight line. The successful fit with the value of *B*_0_ close to an upper critical (crossover) field *B*_c2_(0) = 5.5 T (as shown later) is consistent with the collective creep theory^8,47^.

Figure 1e shows an enlarged view of the MR curves at different *T*. The metal-insulator transition field *B*_MI_ = 5.56 T is given as a field at which all of the MR curves cross and *R*_□_ is independent of *T*. Above *B*_MI_, *R*_□_ shows a logarithmic-like increase with a decrease in *T* as seen in Fig. 1b. As mentioned in our previous report^10^, we regard the weak localization behavior as a character of an insulator^6,10,11^, which is expected to cross over to a strongly insulating state with exponential divergence at much lower temperatures^5,48^. The weakly localized state is also identified as a dirty metal caused by quantum correction^5^. From these results, the *B*-induced SMIT is confirmed in this sample.

Figure 1f displays a Nernst signal *N* measured as a function of *B* at different *T* from 0.1 K to 2.4 K. Figure 1g shows the transverse thermoelectric conductivity *α*_*xy*_ converted from the relation *N* = *R*_□_*α*_*xy*_ (see Methods). For comparison, the MR curve at each *T* is also shown. The *B* and *T* dependences of *N* and *α*_*xy*_ are similar to those reported in vortex systems^10,26,29,33–38^. With increasing *B*, *N* together with *R*_□_ starts to grow above *B*_c_. Then, *N* decreases after showing a peak, which is attributed to a decrease in *α*_*xy*_ (Fig. 1g). The maximum amplitudes of the vortex Nernst signal ${N}_{\max }$ in the *B*-*T* range studied is 2.7 μV/K at 1.2 K in 2.8 T. This value is comparable to ${N}_{\max }$ = 2.9 μV/K of the 12 nm-thick a-Mo_*x*_Ge_1−*x*_ film with *T*_c0_ = 2.58 K used in our previous work^10^ but smaller than ${N}_{\max }$ = 8.3 μV/K of a multi-layered a-Mo_*x*_Ge_1−*x*_ film with *T*_c0_ ~ 6 K^33^. The difference of ${N}_{\max }$ may be due to the difference of effective thickness or dimensionality. In Fig. 2a, b, we plot sensitivity limits of the *N* signals as *B*_*N*→+0_ with open triangles and open inverse triangles in the low and high-*B* regions, respectively.

*α*_*xy*_ consists of three contributions: ${\alpha }_{xy}^{\phi }$ from the mobile vortices in the vortex-liquid state, ${\alpha }_{xy}^{{{{{{{{\rm{fl}}}}}}}}}$ from the amplitude fluctuations, and ${\alpha }_{xy}^{{{{{{{{\rm{n}}}}}}}}}$ from normal electrons^26^. ${\alpha }_{xy}^{\phi }$ is given by ${\alpha }_{xy}^{\phi }={s}_{\phi }/{\phi }_{0}$^26^, where *s*_*ϕ*_ is the transport entropy in the vortex core and *ϕ*_0_( ≡ *h*/2*e*) the flux quantum. According to the theory^49^, with increasing *B* near a crossover field *B*_c2_ in the vortex liquid phase, *s*_*ϕ*_ decreases linearly as *s*_*ϕ*_ ∝ *B*_c2_ − *B* and vanishes at *B*_c2_. Therefore, one can determine *B*_c2_ and distinguish ${\alpha }_{xy}^{\phi }$ in *B* ≤ *B*_c2_ from the other contributions in *B* > *B*_c2_ by linearly extrapolating *α*_*xy*_ to zero as indicated by a solid straight line in Fig. 1g^10,35^. As seen in the high-*B* region (Fig. 1g inset), the contribution of ${\alpha }_{xy}^{{{{{{{{\rm{n}}}}}}}}}$, which increases in proportion to *B*, is negligible in amorphous samples due to a very short mean free path of quasiparticles^10,27–29,38^. Thus, *α*_*xy*_ in *B* > *B*_c2_ corresponds to ${\alpha }_{xy}^{{{{{{{{\rm{fl}}}}}}}}}$.

Recent studies of the vortex Nernst effect have suggested that a maximum value of the vortex transport entropy per unit layer ${s}_{\phi }^{{{{{{{{\rm{sheet}}}}}}}}}$ is of the order of *k*_B_ in many superconductors^33,50,51^. In our experiment, a maximum amplitude of ${\alpha }_{xy}^{\phi }$ = 30 nA/K at 1.6 K in 0.8 T is converted into ${s}_{\phi }={\phi }_{0}{\alpha }_{xy}^{\phi }=4.5{k}_{{{{{{{{\rm{B}}}}}}}}}$. Since *s*_*ϕ*_ represents the vortex transport entropy per film thickness (= 10 nm), ${s}_{\phi }^{{{{{{{{\rm{sheet}}}}}}}}}$ is calculated to be 0.14 *k*_B_ supposing a unit layer thickness of ~ 3 Å for a-Mo_*x*_Ge_1−*x*_. This value is still close to *k*_B_.

We plot *B*_c2_(*T*) thus obtained with solid red squares in the *B*-*T* plane (Fig. 2a–c). Near *T*_c0,*R*_, *B*_c2_(*T*) decreases almost linearly as expected in the Ginzburg-Landau (GL) theory and seems to connect to *T*_c0,*R*_. With decreasing *T*, *B*_c2_(*T*) shows a downward deviation from the linear GL line and saturates to *B*_c2_(*T* → 0) = 5.5 T. The value of *B*_c2_(0) = 5.5 T coincides with *B*_MI_ = 5.56 T within errors, indicating that the insulating phase in this sample corresponds to a Fermi insulator without vortices^4,5^. *B*_c2_(*T*) thus obtained is well reproduced by the Werthamer-Helfand-Hohenberg (WHH) theory^52^ as shown with a dashed line, where the theoretical value of *B*_c2_(0) is given by ${B}_{{{{{{{{\rm{c2}}}}}}}}}(0)=0.69{\widetilde{B}}_{{{{{{{{\rm{c2}}}}}}}}}(0)$ using ${\widetilde{B}}_{{{{{{{{\rm{c2}}}}}}}}}(0)$ ($\equiv {T}_{c0}| {{{{{{{\rm{d}}}}}}}}{B}_{c2}/{{{{{{{\rm{d}}}}}}}}T{| }_{T={T}_{c0}}$) = 8.0 T obtained from a linear extrapolation of *B*_c2_(*T*) in the GL regime to *T* = 0 (Fig. 2c).

Figure 1h, i show the *B* dependence of *N* and *α*_*xy*_, respectively, at different *T* above *T*_c0,*R*_. Both show a nonmonotonic behavior with a peak at a certain field B^*^(*T*). The peak heights are more than one order of magnitude smaller than the ones below *T*_c0,*R*_ (Fig. 1f, g). As *T* increases above *T*_c0,*R*_, B^*^ shifts to higher *B* with a broadening of the peak and a decrease in its height. These behaviors have been observed as common features of ${\alpha }_{xy}^{{{{{{{{\rm{fl}}}}}}}}}$ above *T*_c0,*R*_^26–32^.

According to the theory based on the Gaussian fluctuations^40–44^, ${\alpha }_{xy}^{{{{{{{{\rm{fl}}}}}}}}}$ above *T*_c0,*R*_ in *B* ≪ B^*^ follows the expression ${\alpha }_{xy}^{{{{{{{{\rm{fl}}}}}}}}}=({k}_{{{{{{{{\rm{B}}}}}}}}}{e}^{2}/6\pi {\hslash }^{2}){\xi }_{{{{{{{{\rm{GL}}}}}}}}}^{2}B$, where *ℏ* is the reduced Planck constant, ${\xi }_{{{{{{{{\rm{GL}}}}}}}}}(T)={\xi }_{0}/\sqrt{\ln (T/{T}_{{{{{{{{\rm{c0}}}}}}}}})}$ the GL coherence length, and *ξ*_0_ the Bardeen-Cooper-Schrieffer (BCS) coherence length. This means that the initial slope of ${\alpha }_{xy}^{{{{{{{{\rm{fl}}}}}}}}}(B)$, namely ${\alpha }_{xy}^{{{{{{{{\rm{fl}}}}}}}}}/B{| }_{B\to 0}$, is a measure of *ξ*_GL_(*T*), which is the correlation length of the amplitude fluctuations below B^*^. Thus, a temperature at which ${\alpha }_{xy}^{{{{{{{{\rm{fl}}}}}}}}}/B{| }_{B\to 0}$ diverges corresponds to *T*_c0_ defined from the divergence of *ξ*_GL_(*T*). By plotting the *T* dependence of *α*_*xy*_/*B*∣_*B*→0_ obtained above *T*_c0,*R*_, we can determine *T*_c0_ = 2.36 K (see Supplementary Section III-1), which coincides with *T*_c0,*R*_ = 2.36 K. With increasing *B* at a given *T*, the correlation length of the amplitude fluctuations is reduced from constant *ξ*_GL_(*T*) to a cyclotron radius (magnetic length) ${l}_{B}(B)=\sqrt{\hslash /2eB}$ when *l*_*B*_(*B*) ≤ *ξ*_GL_(*T*)^26,28–32^. Consequently, *α*_*xy*_ exhibits a peak around $B={\phi }_{0}/2\pi {\xi }_{{{{{{{{\rm{GL}}}}}}}}}^{2}(\equiv {B}^{*})$, which is called a ghost (critical) field^26,28–32,44,53^. This means that a temperature at which B^*^(*T*) → 0 with the divergence of *ξ*_GL_(*T*) also corresponds to *T*_c0_. To determine B^*^(*T*), we fit *α*_*xy*_(*B*) in Fig. 1i with an ad hoc fitting function $pB\exp (-q{B}^{r})$ (solid curves)^38^, where *p*, *q*, and *r* are positive fitting parameters. B^*^(*T*) thus obtained is indicated with colored arrows in Fig. 1i and plotted in Fig. 2a–c with solid green squares. B^*^(*T*) approaches zero toward *T*_c0_. The existence of B^*^(*T*) in the normal state is also confirmed by scaling analysis for *α*_*xy*_/*B* (see Supplementary Section III-2).

Figure 2a, b show the contour maps of *N*(*T*, *B*) and *α*_*xy*_(*T*, *B*), respectively. Below the *B*_c2_(*T*) line in the thermal vortex-liquid phase, *N* and *α*_*xy*_ have large magnitudes, which are due to vortex motion. They are observed down to the lowest temperature 0.1 K in the high-*B* range of the AM state, indicating that the AM state originates from the vortex liquid due to the quantum fluctuations, namely quantum vortex liquid (QVL), as reported in our previous work^10^. Meanwhile, above the *B*_c2_(*T*) line, the contribution of the amplitude fluctuations seen from *N* and *α*_*xy*_ is relatively weak but persists in a wide range of the *B*-*T* plane with arc-shaped contour lines (light gray lines).

**Fig. 3:**
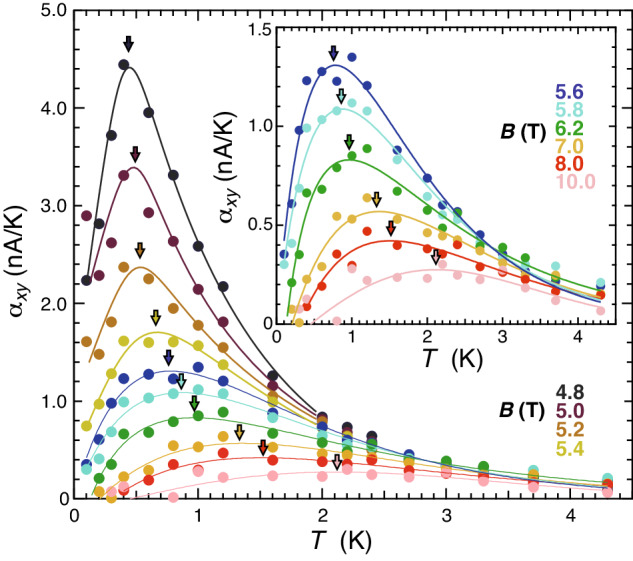
Estimation of a ghost temperature T^*^. The *T* dependences of *α*_*xy*_(*T*) for different *B* from 4.8 T to 10 T. In the inset, *α*_*xy*_(*T*) above *B*_c2_(0) is enlarged. The ghost temperature T^*^(*B*) is estimated from a peak temperature as indicated by colored arrows. It is obtained from the fit lines using an ad hoc function and smoothly connected eye guide lines for the data above and below *B*_c2_(0), respectively, both of which are shown with solid colored curves.

In the main panel of Fig. 3, the *T* dependence of *α*_*xy*_(*T*) is plotted for different *B* from 4.8 T to 10 T. In the inset of Fig. 3, *α*_*xy*_(*T*) above *B*_c2_(0) is enlarged and shown. *α*_*xy*_(*T*) shows a peak at a certain temperature T^*^(*B*). With an increase in *B*, the peak becomes broad, its height decreases, and T^*^ increases. These behaviors closely resemble those of theoretically obtained ${\alpha }_{xy}^{{{{{{{{\rm{fl}}}}}}}}}(T)$ above *B*_c2_(0) (Fig. 2b in ref. ^44^), where the peak temperature T^*^(*B*) called a ghost temperature separates the thermal and quantum regimes of the amplitude fluctuations. A similar thermal-to-quantum crossover temperature has been theoretically predicted^41–43,54^. Although a seemingly similar behavior was observed earlier in the high-*B* region of cuprates^36^, its origin was different. It was interpreted as originating from vortices with large quantum fluctuations^36,39^.

According to the theory^43^, the quantum amplitude fluctuations below T^*^(*B*) are described as puddle-like fluctuations with a size of the quantum correlation length ${\xi }_{{{{{{{{\rm{qf}}}}}}}}}(B)={\xi }_{0}/\sqrt{\ln (B/{B}_{{{{{{{{\rm{QCP}}}}}}}}})}$, which diverges at a QCP denoted by *B*_QCP_ (see Supplementary Section III-3). In our MR data (Fig. 1e), indeed, the existence of the quantum amplitude fluctuations is suggested by a slight hump around *B*_*p*_ ~ 6.5−7.0 T ( > *B*_c2_(0)) near *T* = 0 (*T* = 0.07−0.14 K)^5,43^, as found in our previous report^10^. Furthermore, the theories^41–44^ show that ${\alpha }_{xy}^{{{{{{{{\rm{fl}}}}}}}}}(T)$ above *B*_c2_(0) at *T* ≪ T^*^ is proportional to ${\xi }_{{{{{{{{\rm{qf}}}}}}}}}^{2}T$ (see Supplementary Section III-3). This means that an initial slope of ${\alpha }_{xy}^{{{{{{{{\rm{fl}}}}}}}}}(T)$, namely ${\alpha }_{xy}^{{{{{{{{\rm{fl}}}}}}}}}/T{| }_{T\to 0}$, at given *B* is a measure of *ξ*_qf_(*B*). Therefore, a field at which ${\alpha }_{xy}^{{{{{{{{\rm{fl}}}}}}}}}/T{| }_{T\to 0}$ diverges with T^*^(*B*) → 0 corresponds to a QCP determined from the amplitude fluctuations. To determine T^*^, we fit *α*_*xy*_(*T*) above *B*_c2_(0) with a function $p(T-{T}_{0})\exp (-q{T}^{r})$ (colored solid lines) similar to the one mentioned above, where *T*_0_ corresponds to the sensitivity limit of *α*_*xy*_ at *B*_*N*→+0_. For *α*_*xy*_(*T*) below *B*_c2_(0), we draw eye-guide lines (colored solid lines). T^*^(*B*) thus obtained is indicated with colored arrows in the main panel and the inset of Fig. 3 and plotted in Fig. 2a–c with solid blue squares. With decreasing *B*, T^*^(*B*) approaches zero toward a field below *B*_c2_(0). This indicates that the location of *B*_QCP_ determined from the amplitude fluctuations is inside the AM state. Thus, *B*_MI_( ≈ *B*_c2_(0)) is not a QCP but a crossover point, below which the quantum amplitude fluctuations are transformed into the QVL. To our knowledge, this is the first experiment to determine the ghost temperature T^*^(*B*) in the *B*-*T* plane and the QCP inside the AM state. The existence of T^*^(*B*) is also confirmed by scaling analysis for *α*_*xy*_/*T* (see Supplementary Section III-4).

Figure 2d shows the contour map of the theoretical values of ${\alpha }_{xy}^{{{{{{{{\rm{fl}}}}}}}}}(T,B)(\equiv {\alpha }_{xy}^{{{{{{{{\rm{fl}}}}}}}},{{{{{{{\rm{theo}}}}}}}}}(T,B))$ calculated for a 2D superconductor based on the Gaussian fluctuations^42–44^ using the codes provided in ref. ^43^. Here, we input only two parameters, *T*_c0_ = 2.36 K and *B*_c2_(0) = 5.5 T obtained experimentally. The *T* and *B* axes are normalized by *T*_c0_ and ${\widetilde{B}}_{{{{{{{{\rm{c2}}}}}}}}}(0)$, respectively. A theoretical line of *B*_c2_(*T*) (a dashed line), which is drawn by the WHH theory^52^, is identical to the fitting line of the experimental data of *B*_c2_(*T*) (a dashed line in Fig. 2a–c). It is seen from the contour maps that the theoretical values of ${\alpha }_{xy}^{{{{{{{{\rm{fl}}}}}}}},{{{{{{{\rm{theo}}}}}}}}}(T,B)$ in Fig. 2d reproduce almost quantitatively the experimental data of ${\alpha }_{xy}^{{{{{{{{\rm{fl}}}}}}}}}(T,B)$ in Fig. 2b, both of which persist in the wide range of the *B*-*T* plane above *B*_c2_(*T*). The green and blue lines in Fig. 2c, d represent the theoretical lines of ${B}^{*}(T)(\equiv {B}_{{{{{{{{\rm{theo}}}}}}}}}^{*}(T))$ and ${T}^{*}(B)(\equiv {T}_{{{{{{{{\rm{theo}}}}}}}}}^{*}(B))$ obtained from the *B* and *T* dependences of ${\alpha }_{xy}^{{{{{{{{\rm{fl}}}}}}}},{{{{{{{\rm{theo}}}}}}}}}(T,B)$, respectively. It is found that the experimental data of B^*^(*T*) (green squares) in Fig. 2c are in good agreement with the theoretical values of ${B}_{{{{{{{{\rm{theo}}}}}}}}}^{*}(T)$ (a green line) within error bars. By contrast, the experimental data of T^*^(*B*) (blue squares) in Fig. 2c clearly fall on a line below the theoretical line of ${T}_{{{{{{{{\rm{theo}}}}}}}}}^{*}(B)$ (a blue line). However, when ${T}_{{{{{{{{\rm{theo}}}}}}}}}^{*}(B)$ is vertically compressed by a factor of 4.6/5.5 as shown with an orange line ($\equiv {T}_{{{{{{{{\rm{theo}}}}}}}},{{{{{{{\rm{m}}}}}}}}}^{*}(B)$), the experimental data of T^*^(*B*) well fall on the modified theoretical line of ${T}_{{{{{{{{\rm{theo}}}}}}}},{{{{{{{\rm{m}}}}}}}}}^{*}(B)$ (see also Supplementary Section II). From the end point of the orange line, ${T}_{{{{{{{{\rm{theo}}}}}}}},{{{{{{{\rm{m}}}}}}}}}^{*}(B\to {B}_{{{{{{{{\rm{QCP}}}}}}}}})\to 0$, marked with a solid orange circle, *B*_QCP_ = 4.6 T is determined. To our knowledge, this value is first obtained from the present Nernst-effect measurements that probe the amplitude fluctuations, which could not be obtained from conventional measurements, such as resistance. Furthermore, the criticality at *B*_QCP_ is also confirmed from the *B* dependence of ${\alpha }_{xy}^{{{{{{{{\rm{fl}}}}}}}}}/T{| }_{T\to 0}(\propto {\xi }_{{{{{{{{\rm{qf}}}}}}}}}{(B)}^{2})$ above *B*_c2_(0), which shows a divergent behavior toward *B*_QCP_ (see Supplementary Section III-3).

## Discussion

**Fig. 4:**
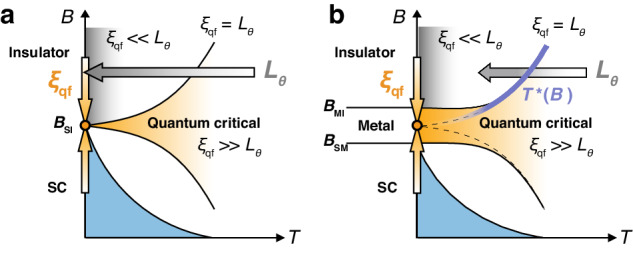
Schematic models of quantum criticality in SIT and SMIT. **a** A standard picture of QPTs for the *B*-induced SIT in the *B*-*T* plane^1 --3^. The critical field *B*_SI_ of the SIT corresponds to the QCP at *T* = 0. **b** A proposed model of quantum criticality in the SMIT^10,20^. The AM state stems from broadening of the SIT, which is verified in this work by finding *B*_QCP_ (solid orange circle) as a field that the experimental T^*^(*B*) line approaches as indicated by a solid blue line. In both (**a**, **b**), the quantum critical regime is defined by the area where *ξ*_qf_(*B*) > *L*_*θ*_(*T*). Gray and orange arrows indicate the direction of the increase in *L*_*θ*_(*T*) and *ξ*_qf_(*B*), respectively.

Finally, let us discuss the origin of the AM state. Generally, the quantum critical regime is defined by *ξ*_qf_(*B*) > *L*_*θ*_(*T*)^1–3^, where *L*_*θ*_(*T*) ~ *T*^−1/*z*^ is a dephasing length and *z* is a dynamical critical exponent^1^. In the standard QPTs including the SIT, with decreasing *T*, the field range of the quantum critical regime is reduced to zero toward the QCP as illustrated in Fig. 4a because *L*_*θ*_(*T*) diverges as *T* → 0. However, if *L*_*θ*_(*T*) is saturated to a constant value below a certain temperature *T*_sat_ for some reasons^55^, the field range satisfying *ξ*_qf_(*B*) > *L*_*θ*_(*T*_sat_) remains nonzero at *T* = 0 as in Fig. 4b^20^, resulting in the quantum critical ground state that spans over an extended field range. Indeed, the saturation of *L*_*θ*_(*T*) has been observed in the AM state of a JJA^16^. In our experiment, the existence of the QCP is clearly detected as *B*_QCP_ inside the AM state. Moreover, the quantum critical behavior^37,46,56^ is observed not only at *B*_QCP_ but also in the wide range of *B* inside the AM state in the present sample (see Supplementary Fig. S4) as well as in our previous report^10^. From these results, we conclude that the AM state originates from the broadening of the SIT. This is consistent with the theoretical prediction^19^ that there exists a broadened quantum ground state between *B*_SM_ and *B*_MI_. It is likely that the thermal-to-quantum crossover (ghost-temperature) line T^*^(*B*) obtained in this work reflects a boundary where *ξ*_qf_(*B*) = *L*_*θ*_(*T*) above *T*_sat_ (see Supplementary Section III-4).

A possible origin of the saturation of *L*_*θ*_(*T*) in superconductors is a coupling between Cooper pairs and a fermionic dissipative bath, which makes the pure SIT unstable^9,15–17,20,57^. Meanwhile, in magnetic materials, the emergence of the quantum critical phase is attributed to magnetic frustration, leading to a possible quantum spin liquid^23,24^. Indeed, ref. ^19^ theoretically suggests that the critical AM state has characteristics analogous to the quantum spin liquid. Thus, some frustration effect on the Cooper pairs from a dissipative bath can be an origin of the AM state.

To conclude, by constructing the thorough contour map of the Nernst signal in the *B*-*T* plane, we reveal an evolution of the superconducting fluctuations in the disordered 2D superconductor exhibiting the SMIT. We found the thermal-to-quantum crossover line (ghost-temperature line) T^*^(*B*) associated with the QPT, as well as the ghost-field line B^*^(*T*) associated with a thermal transition. The *T* = 0 QCP determined from T^*^(*B* → *B*_QCP_) → 0 is located inside the AM state, indicating that the AM state is a broadened critical state of the SIT. Our work also shows the usefulness of the Nernst effect to study QPTs in superconducting systems.

## Methods

### Samples

An a-Mo_*x*_Ge_1−*x*_ film with *x* = 0.77 and a thickness of 10 nm was prepared by rf-sputtering onto a 0.15 mm thick glass substrate. The film thickness is comparable to *ξ*_0_ ~ 8 nm estimated from ${\xi }_{0}^{2}={\phi }_{0}/(2\pi {B}_{{{{{{{{\rm{c}}}}}}}}2}(0))$ with *B*_c2_(0) = 5.5 T. To obtain a homogeneous amorphous film, the substrate was mounted on a water-cooled holder rotating with 240 rpm during sputtering. Ag electrodes were deposited 4.2 mm (= *L*) and 5.6 mm (= *l*) apart for longitudinal *V*_*x*_ and transverse voltage *V*_*y*_, respectively. The film thus prepared was protected by depositing a thin SiO layer. *T*_c0,*R*_ = 2.36 K and *T*_c_ = 1.40 K are determined from *R*_□_(*T*_c0,*R*_) = 0.5*R*_□,n_ = 0.5*R*_□_(3.0 K) and the resistance drop below the measurement limit, respectively. *T*_c_ = 1.40 K is reduced compared with *T*_c_ = 6.2 K for the 330 nm thick film^58^. The broadened transition curve indicates 2D nature of superconductivity, similarly to the 12 nm thick film with *T*_c_ = 2.13 K used in our previous work^10^.

### Resistivity and Nernst-effect measurements

All measurements were carried out using a sample holder installed in a dilution refrigerator^10^. The magnetic field *B* was applied perpendicular to the film plane. *R*_□_ = (*V*_*x*_/*L*)/(*I*_*x*_/*l*) was obtained using standard four-terminal dc and low-frequency ac (19 Hz) lock-in methods with a bias current of *I*_*x*_≥ 30 nA in the ohmic regime.

*N* was obtained from *N* ≡ *E*_*y*_/ ∇ *T*_*x*_ = (*V*_*y*_/*l*)/(Δ*T*/*L*), where Δ*T* = *T*_high_ − *T*_low_ is a temperature difference. *V*_*y*_ was measured with a nanovoltmeter. To set Δ*T*, a heat current was applied from a heater on one side of the glass substrate to the other side glued on a heat bath. Δ*T* was measured by two RuO_2_ thermometers, which were thermally isolated from the bath but thermally coupled to the two Ag electrodes of *V*_*x*_ through Cu wires. An average sample temperature was defined by *T*_ave_ = (*T*_high_ + *T*_low_)/2. Δ*T* was fixed to be 30%, ~20%, and ~ 10 % of *T*_ave_ at 0.1 K, 0.2−0.6 K, and 0.8−4.3 K, respectively, to obtain measurable *V*_*y*_ keeping Δ*T* as low as possible. At each measurement step, we switched on and off Δ*T* to subtract the background thermoelectric voltage observed at Δ*T* = 0 from *V*_*y*_ at Δ*T* ≠ 0. To correct misalignment of heat flow, we extracted an antisymmetric contribution of *N* with respect to magnetic field reversal. *N* from the superconducting fluctuations is approximated by *N* = *R*_□_*α*_*xy*_ due to the particle-hole symmetry^40,59^. *α*_*xy*_ is a thermodynamic quantity proportional to the transport entropy, while *R*_□_ reflects electron scattering and viscosity of vortices.

We checked an effect of external noise using low-pass RC filters with a cutoff frequency of 100 kHz, which is adequate to eliminate the noise effect^21,22^. Although the filters were inserted between the sample and a nanovoltmeter as well as between the sample and a current bias source at room temperature, the results with and without the filters were almost the same. Note that, in the Nernst-effect measurements, the sample was electrically isolated from the current bias source, which is a main possible origin of external noise^21,22^.

### Supplementary information

Supplementary Information

Peer Review File

### Source data

Source Data

## Data Availability

The data used in this study are available in the main text and the Supplementary Information. All other data are available from the corresponding author upon request. Source data are provided with this paper.

## References

[1] Sondhi SL, Girvin SM, Carini J, Shahar D (1997). Continuous quantum phase transitions. Rev. Mod. Phys..

[2] Vojta M (2003). Quantum phase transitions. Rep. Prog. Phys..

[3] Löhneysen HV, Rosch A, Vojta M, Wölfle P (2007). Fermi-liquid instabilities at magnetic quantum phase transitions. Rev. Mod. Phys..

[4] Goldman AM, Markovic N (1998). Superconductor-insulator transitions in the two-dimensional limit. Phys. Today.

[5] Gantmakher VF, Dolgopolov VT (2010). Superconductor–insulator quantum phase transition. Phys. Usp..

[6] Okuma S, Terashima T, Kokubo N (1998). Anomalous magnetoresistance near the superconductor-insulator transition in ultrathin films of a-Mo_*x*_Si_1−*x*_. Phys. Rev. B.

[7] Lee PA, Ramakrishnan TV (1985). Disordered electronic systems. Rev. Mod. Phys..

[8] Ephron D, Yazdani A, Kapitulnik A, Beasley MR (1996). Observation of quantum dissipation in the vortex state of a highly disordered superconducting thin film. Phys. Rev. Lett..

[9] Mason N, Kapitulnik A (1999). Dissipation effects on the superconductor-insulator transition in 2D superconductors. Phys. Rev. Lett..

[10] Ienaga K, Hayashi T, Tamoto Y, Kaneko S, Okuma S (2020). Quantum criticality inside the anomalous metallic state of a disordered superconducting thin film. Phys. Rev. Lett..

[11] Zhang X, Hen B, Palevski A, Kapitulnik A (2021). Robust anomalous metallic states and vestiges of self-duality in two-dimensional granular In-InO_*x*_ composites. npj Quantum Mater..

[12] Saito Y, Kasahara Y, Ye J, Iwasa Y, Nojima T (2015). Metallic ground state in an ion-gated two-dimensional superconductor. Science.

[13] Sharma CH, Surendran AP, Varma SS, Thalakulam M (2018). 2D superconductivity and vortex dynamics in 1T-MoS_2_. Commun. Phys..

[14] Xing Y (2021). Extrinsic and intrinsic anomalous metallic states in transition metal dichalcogenide Ising superconductors. Nano Lett..

[15] Bøttcher CGL (2018). Superconducting, insulating and anomalous metallic regimes in a gated two-dimensional semiconductor–superconductor array. Nat. Phys..

[16] Yang C (2019). Intermediate bosonic metallic state in the superconductor-insulator transition. Science.

[17] Yang C (2022). Signatures of a strange metal in a bosonic system. Nature.

[18] Shimshoni E, Auerbach A, Kapitulnik A (1998). Transport through quantum melts. Phys. Rev. Lett..

[19] Das D, Doniach S (1999). Existence of a Bose metal at *T*= 0. Phys. Rev. B.

[20] Kapitulnik A, Mason N, Kivelson SA, Chakravarty S (2001). Effects of dissipation on quantum phase transitions. Phys. Rev. B.

[21] Tamir I (2019). Sensitivity of the superconducting state in thin films. Sci. Adv..

[22] Dutta S (2019). Extreme sensitivity of the vortex state in a-MoGe films to radio-frequency electromagnetic perturbation. Phys. Rev. B.

[23] Zhao H (2019). Quantum-critical phase from frustrated magnetism in a strongly correlated metal. Nat. Phys..

[24] Suzuki Y (2022). Mott-Driven BEC-BCS Crossover in a Doped Spin Liquid Candidate *κ* − (BEDT−TTF)_4_Hg_2.89_Br_8_. Phys. Rev. X.

[25] Shi X, Lin PV, Sasagawa T, Dobrosavljević V, Popović D (2014). Two-stage magnetic-field-tuned superconductor–insulator transition in underdoped La_2−*x*_Sr_*x*_CuO_4_. Nat. Phys..

[26] Behnia K, Aubin H (2016). Nernst effect in metals and superconductors: a review of concepts and experiments. Rep. Prog. Phys..

[27] Pourret A (2006). Observation of the Nernst signal generated by fluctuating Cooper pairs. Nat. Phys..

[28] Pourret A (2007). Length scale for the superconducting Nernst signal above *T*_*c*_ in Nb_0.15_Si_0.85_. Phys. Rev. B.

[29] Pourret A, Spathis P, Aubin H, Behnia K (2009). Nernst effect as a probe of superconducting fluctuations in disordered thin films. N. J. Phys..

[30] Chang J (2012). Decrease of upper critical field with underdoping in cuprate superconductors. Nat. Phys..

[31] Yamashita T (2015). Colossal thermomagnetic response in the exotic superconductor URu_2_Si_2_. Nat. Phys..

[32] Mandal PR, Sarkar T, Higgins JS, Greene RL (2018). Nernst effect in the electron-doped cuprate superconductor La_2−*x*_Ce_*x*_CuO_4_. Phys. Rev. B.

[33] Rischau CW (2021). Universal bound to the amplitude of the vortex Nernst signal in superconductors. Phys. Rev. Lett..

[34] Xu ZA, Ong NP, Wang Y, Kakeshita T, Uchida S (2000). Vortex-like excitations and the onset of superconducting phase fluctuation in underdoped La_2−*x*_Sr_*x*_CuO_4_. Nature.

[35] Wang Y (2002). High field phase diagram of cuprates derived from the Nernst effect. Phys. Rev. Lett..

[36] Capan C, Behnia K, Li ZZ, Raffy H, Marin C (2003). Anomalous dissipation in the mixed state of underdoped cuprates close to the superconductor-insulator boundary. Phys. Rev. B.

[37] Izawa K (2007). Thermoelectric response near a quantum critical point: the case of CeCoIn_5_. Phys. Rev. Lett..

[38] Roy A, Shimshoni E, Frydman A (2018). Quantum criticality at the superconductor-Insulator transition probed by the Nernst effect. Phys. Rev. Lett..

[39] Ikeda R (2002). Fluctuation effects in underdoped cuprate superconductors under a magnetic field. Phys. Rev. B.

[40] Ussishkin I, Sondhi SL, Huse DA (2002). Gaussian superconducting fluctuations, thermal transport, and the Nernst effect. Phys. Rev. Lett..

[41] Michaeli K, Finkel’stein AM (2009). Quantum kinetic approach to the calculation of the Nernst effect. Phys. Rev. B.

[42] Serbyn MN, Skvortsov MA, Varlamov AA, Galitski V (2009). Giant Nernst effect due to fluctuating Cooper pairs in superconductors. Phys. Rev. Lett..

[43] Varlamov AA, Galda A, Glatz A (2018). Fluctuation spectroscopy: from Rayleigh-Jeans waves to Abrikosov vortex clusters. Rev. Mod. Phys..

[44] Glatz A, Pourret A, Varlamov AA (2020). Analysis of the ghost and mirror fields in the Nernst signal induced by superconducting fluctuations. Phys. Rev. B.

[45] Sergeev A, Reizer M (2021). Entropy-based theory of thermomagnetic phenomena. Int. J. Mod. Phys. B.

[46] Machida Y (2012). Thermoelectric response near a quantum critical point of *β*-YbAlB_4_ and YbRh_2_Si_2_: a comparative study. Phys. Rev. Lett..

[47] Feigel’man MV, Geshkenbein VB, Larkin AI (1990). Pinning and creep in layered superconductors. Phys. C..

[48] Liu Y, Haviland DB, Nease B, Goldman AM (1993). Insulator-to-superconductor transition in ultrathin films. Phys. Rev. B.

[49] Maki K (1968). Thermomagnetic effects in dirty type-II superconductors. Phys. Rev. Lett..

[50] Huebener RP, Ri H-C (2021). Vortex transport entropy in cuprate superconductors and Boltzmann constant. Phys. C..

[51] Behnia K (2023). Nernst response, viscosity and mobile entropy in vortex liquids. J. Phys.: Condens. Matter.

[52] Werthamer NR, Helfand E, Hohenberg PC (1966). Temperature and purity dependence of the superconducting critical field, *H*_*c*2_. III. Electron spin and spin-orbit effects. Phys. Rev..

[53] Kapitulnik A, Palevski A, Deutscher G (1985). Inhomogeneity effects on the magnetoresistance and the ghost critical field above *T*_c_ in thin mixture films of In-Ge. J. Phys. C: Solid State Phys..

[54] Ishida H, Ikeda R (2002). Theoretical description of resistive behavior near a quantum vortex-glass transition. J. Phys. Soc. Jpn..

[55] Lin JJ, Bird JP (2002). Recent experimental studies of electron dephasing in metal and semiconductor mesoscopic structures. J. Phys.: Cond. Matt..

[56] Poran S (2017). Quantum criticality at the superconductor-insulator transition revealed by specific heat measurements. Nat. Commun..

[57] Zhu L, Chen Y, Varma CM (2015). Local quantum criticality in the two-dimensional dissipative quantum XY model. Phys. Rev. B.

[58] Okuma S, Kashiro K, Suzuki Y, Kokubo N (2008). Order-disorder transition of vortex matter in a-Mo_*x*_Ge_1−*x*_ films probed by noise. Phys. Rev. B.

[59] Wang Y (2001). Onset of the vortexlike Nernst signal above *T*_*c*_ in La_2−*x*_Sr_*x*_CuO_4_ and Bi_2_Sr_2−*y*_La*y*CuO_6_. Phys. Rev. B.

