# Supplementary material for: Broadened quantum critical ground state in a disordered superconducting thin film
Source: Nat Commun. 2024 Mar 16;15:2388. doi: 10.1038/s41467-024-46628-7 (PMC10944498; doi:10.1038/s41467-024-46628-7)
Supplement: Supplementary file 1 — Supplementary Information [file 41467_2024_46628_MOESM1_ESM.pdf]

Supplementary information: Broadened quantum critical ground state in a disordered superconducting thin film

Koichiro Ienaga,[1,*] Yutaka Tamoto,[1] Masahiro Yoda,[1]
Yuki Yoshimura,[1] Takahiro Ishigami,[1] and Satoshi Okuma[1]

[1] *Department of Physics, Tokyo Institute of Technology,*
2-12-1 Ohokayama, Meguro-ku, Tokyo 152-8551 Japan

(Dated: February 15, 2024)

* ienaga.k.aa@m.titech.ac.jp

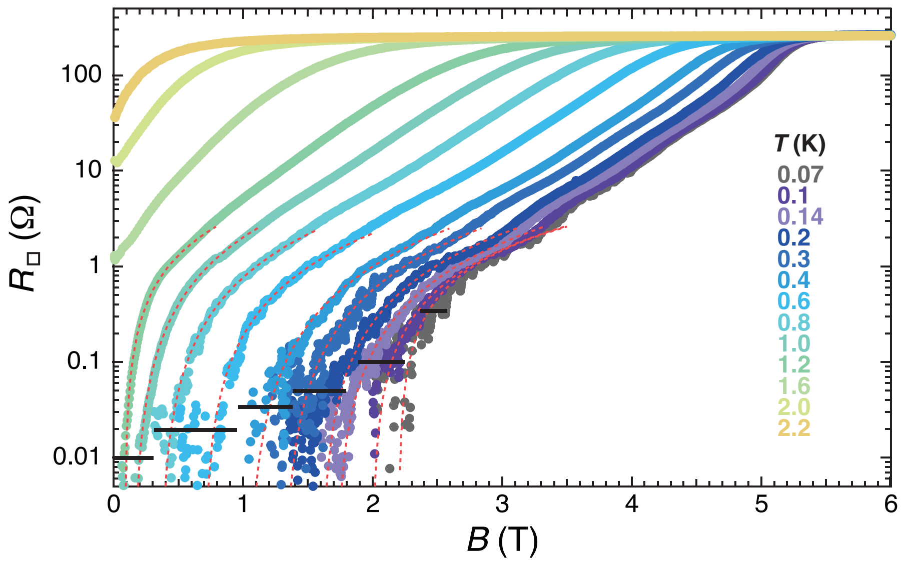

Fig. S1. **The B dependence of $R_\square$ at different T.** Current biases were changed depending on the T range to suppress the possible heating effect: 30 nA at 0.07 K, 100 nA at 0.1 and 0.14 K, 200 nA at 0.2 and 0.3 K, 300nA at 0.4 K, 500 nA at 0.6 and 0.8 K, and 1.0 μA at $T \geq 1.0$ K. The sensitivity limit is indicated by horizontal bars. Power-law fits with $R_\square \sim (B - B_c)^\mu$ are shown by dashed red curves.

I. Determination of the boundary of the vortex-glass (VG) phase

Figure S1 shows the magnetoresistance (MR) at fixed temperatures T. With increasing field B, the sheet resistance $R_\square$ rises from zero in a power-law fashion $R_\square \sim (B - B_c)^\mu$ and then increases in an exponential-like fashion $R_\square \sim \exp(B/\lambda)$, where B_c is a zero resistance field corresponding to a boundary between the VG phase and the vortex-liquid phase, and μ and λ are positive fitting parameters. These behaviors are good agreement with the previous result in amorphous (a-)Mo_xGe_{1-x} thin films [1]. To determine B_c, thus, we fit our MR data using the above power-law formula as shown with dashed red lines. We obtain reasonable fits with $\mu \sim 1.2 - 1.9$, which is close to $\mu \sim 1$ observed in the previous study [1]. The extracted values of B_c are plotted in Fig. 2a,b in the main text. The existence of the VG phase at low but nonzero temperatures was also reported in our previous studies of a-Mo_xGe_{1-x} thin films with a thicknesses of 10 nm [2] and 12 nm [3], while the VG theory for two-dimensional (2D) superconductors predicts the presence of the VG phase only at $T = 0$ [4].

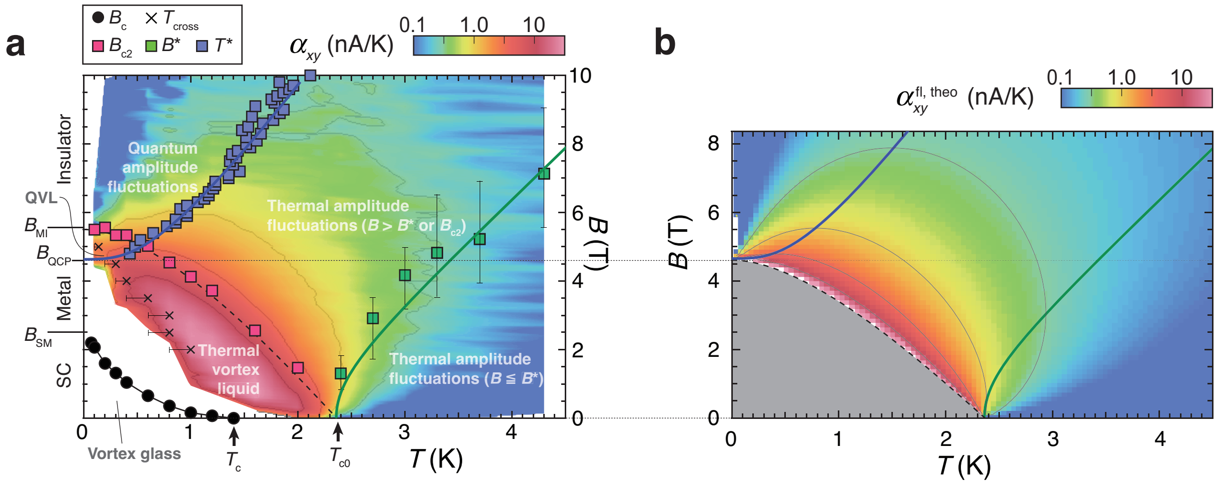

Fig. S2. **Comparison of an experimental contour map of $\alpha_{xy}(T, B)$ with a modified theoretical one with an improved parameter.** **a**, A contour map of experimentally obtained $\alpha_{xy}(T, B)$ (a copy of Fig. 2b) in the B-T plane, where $B_{c2}(0) = 5.5$ T and $B_{QCP} = 4.6$ T. Green and blue lines indicate modified theoretical lines of $B^*(T)(\equiv B^*_{\mathrm{theo,m}}(T))$ and $T^*(B)(\equiv T^*_{\mathrm{theo,m}}(B))$, respectively, calculated using an improved parameter (see **b**). Error bars for B^* represent B ranges in which $\alpha_{xy}(B)$ at fixed T exceeds 95 % of the peak amplitude. **b**, A modified contour map of theoretically obtained $\alpha^{\mathrm{fl}}_{xy}(T, B)(\equiv \alpha^{\mathrm{fl,theo}}_{xy}(T, B))$ calculated for a 2D superconductor based on the Gaussian fluctuations [5, 6] using $B_{c2}(0) = 4.6$ T instead of 5.5 T. The green and blue lines represent $B^*_{\mathrm{theo,m}}(T)$ and $T^*_{\mathrm{theo,m}}(B)$ lines, respectively.

II. The quantum critical point identified by the amplitude fluctuations

As mentioned in the main text, while the experimental contour map of the transverse thermoelectric conductivity $\alpha_{xy}(T, B)$ (Fig. 2b) is reproduced almost quantitatively by the theoretical one (Fig. 2d) [5, 6], the ghost temperature line $T^*(B)$ obtained experimentally shifts downward from the theoretical line of $T^*(B)(\equiv T^*_{\mathrm{theo}}(B))$. This is because for the calculation, the input parameter of the critical field is $B_{c2}(0)$ ($= 5.5$ T), which is different from the actual critical field of B_{QCP} ($=$ 4.6 T) determined from the amplitude fluctuations. Thus, we compare the experimental result of $\alpha_{xy}(T, B)$ (Fig. S2a, a copy of Fig. 2b) with the theoretical result of $\alpha^{\mathrm{fl}}_{xy}(T, B)(\equiv \alpha^{\mathrm{fl,theo}}_{xy}(T, B))$ for the amplitude fluctuations calculated with $B_{c2}(0) = 4.6$ T (Fig. S2b), where green and blue lines are modified theoretical lines of $B^*(T)(\equiv B^*_{\mathrm{theo,m}}(T))$ and $T^*(B)(\equiv T^*_{\mathrm{theo,m}}(B))$, respectively.

The two contour maps show better agreement with each other, compared with the two contour maps shown in Fig. 2b,d. The experimental data point of $T^*(B)$ shown with blue squares in Fig. S2a well fall on the $T^*_{\mathrm{theo,m}}(B)$ line shown here, which is the same as the orange line in Fig. 2c. Also, the $B^*_{\mathrm{theo,m}}(T)$ line, which is 4.6/5.5 times smaller than the $B^*_{\mathrm{theo}}(T)$ line in Fig. 2c, shows good agreement with the experimental data of $B^*(T)$ shown with green squares in Fig. S2a within error bars. These results clearly indicate that the theory [5, 6] for the amplitude fluctuations can be well applied to analyze the data of $\alpha_{xy}(T, B)$ and that $B_{QCP} = 4.6$ T ($< B_{c2}(0)$) is deduced accurately from the extrapolation of the $T^*_{\mathrm{theo,m}}(B)$ line.

III. Critical behaviors and correlation lengths in $\alpha_{xy}(T, B)$

1. Thermal critical behavior in $\alpha_{xy}(T, B)$

As mentioned in the main text, according to the theory based on the Gaussian fluctuations, the contribution of the amplitude fluctuations to α_{xy}, denoted as $\alpha_{xy}^{\rm fl}$, is given in the Ginzburg-Landau (GL) regime ($0 < \ln(T/T_{\rm c0}) \ll 1$, $B \ll B^*$) by [7]

$$\frac{\alpha_{xy}^{\rm fl}}{B} = (k_{\rm B}e^2/6\pi\hbar^2)\xi_{\rm GL}^2 \propto \xi_{\rm GL}^2, \tag{1}$$

where k_B is the Boltzmann constant, $\hbar$ the reduced Planck constant, $\xi_{\rm GL}(T) = \xi_0/\sqrt{\ln(T/T_{\rm c0})}$ the GL correlation length, and ξ_0 the Bardeen-Cooper-Schrieffer (BCS) coherence length. This means that α_{xy}/B is independent of B in the low-B region ($B \ll B^*$) and an initial slope of $\alpha_{xy}^{\rm fl}(B)$ at fixed T, i.e., $\alpha_{xy}^{\rm fl}/B|_{B\to 0}$, is a measure of $\xi_{\rm GL}(T)$, which diverges at $T_{\rm c0}$. For arbitrary T above $T_{\rm c0}$ including T outside the GL regime, Eq. (1) is modified in the low-B region ($B \ll B^*$) as [8, 9]

$$\frac{\alpha_{xy}^{\rm fl}}{B} = \frac{e^2 D}{6\pi^2\hbar}\frac{1}{T\ln(T/T_{\rm c0})} \propto \frac{\xi_{\rm GL}^2}{T}, \tag{2}$$

where $D = 4k_{\rm B}T_{\rm c0}/\pi e\widetilde{B}_{\rm c2}(0)$ is the diffusion coefficient and $\widetilde{B}_{\rm c2}(0) \equiv T_{\rm c0}|{\rm d}B_{\rm c2}/{\rm d}T|_{T=T_{\rm c0}}$ is a linear extrapolation of $B_{\rm c2}(T)$ in the GL regime to $T = 0$. For our data, we experimentally obtained $\widetilde{B}_{\rm c2}(0) = 8.0$ T as shown with a dotted red line in Fig. 2c.

In Fig. S3a, we display the B dependence of α_{xy}/B at different T, which is converted from Fig. 1g,i. Above $T_{\rm c0}(= 2.36$ K), where $\alpha_{xy}(B)$ is attributed to $\alpha_{xy}^{\rm fl}(B)$, α_{xy}/B exhibits saturation to a constant value in the low-B region, giving the value of $\alpha_{xy}^{\rm fl}/B|_{B\to 0}$. In Fig. S3c, we plot $\alpha_{xy}/B|_{B\to 0}$ above $T_{\rm c0}$ extracted from Fig. S3a as a function of $(T/T_{\rm c0})\ln(T/T_{\rm c0})$ for different values of $T_{\rm c0}$ on a log-log scale, where a solid straight line corresponds to Eq. (2). The best fit is obtained with $T_{\rm c0}$ = 2.36 K. It is noted that the plot of $\alpha_{xy}/B|_{B\to 0}$ with $T_{\rm c0} = 2.36$ K is not only in proportion to $1/(T/T_{\rm c0})\ln(T/T_{\rm c0})$ but also in quantitative agreement with Eq. (2). This is surprising, considering that the fitting parameter is only $T_{\rm c0}$. Such quantitative fit for the experimental results using the single fitting parameter $T_{\rm c0}$ has been reported in some previous works [8–12]. Thus, we were able to determine $T_{\rm c0} = 2.36$ K from the thermodynamic quantity α_{xy}. This value coincides with $T_{{\rm c0},R} = 2.36$ K defined from the resistance measurement. These results indicate that criticality at $T_{\rm c0}$, which appears associated with the amplitude fluctuations in the thermal regime, is suitably probed by means of α_{xy}.

In the theory based on the Gaussian fluctuations, $T_{\rm c0}$ is a critical temperature. Meanwhile, for actual 2D superconductors, $T_{\rm c0}$ is a crossover temperature from the amplitude fluctuation regime ($T > T_{\rm c0}$) to the Berezinskii-Kosterlitz-Thouless (BKT) fluctuation regime ($T_{\rm c} < T \leq T_{\rm c0}$). With decreasing T, the amplitude fluctuations tend to vanish toward $T_{\rm c0}$, but do not completely vanish at $T_{\rm c0}$ and marge into the phase fluctuations in the BKT fluctuation regime below $T_{\rm c0}$ [13]. Even in such a situation, application of the Gaussian fluctuations to 2D superconductors is justified except just above $T_{\rm c0}$ as discussed in Ref. 11.

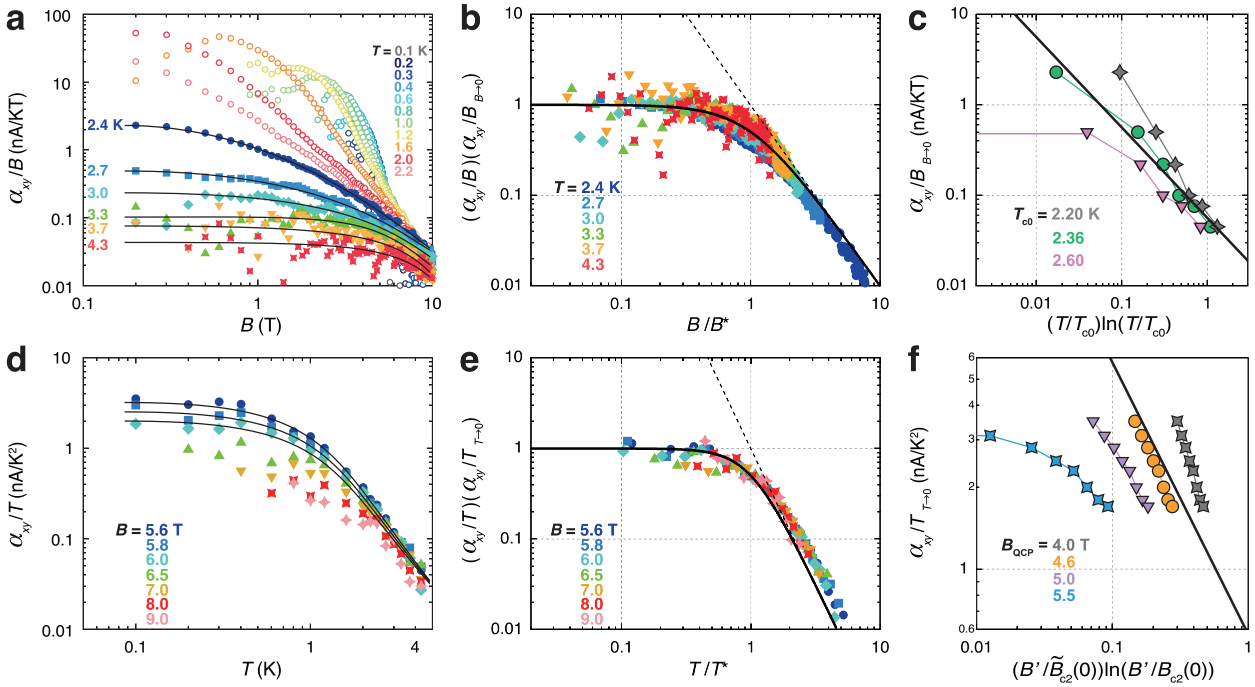

Fig. S3. **Thermal and quantum critical behavior of α_{xy} in the Gaussian fluctuation regime.** **a**, The B dependence of α_{xy}/B at different T converted from Fig. 1g,i. Solid black lines are guides to the eye. **b**, α_{xy}/B above T_{c0} in **a** is divided by $\alpha_{xy}/B|_{B\to 0}$, a saturated value in the low-B region, and plotted against B/B^*. All of the data at different T collapse on a solid black line given by Eq. (4). A dashed straight line indicates a power-law relation in the high-B region represented by Eq. (3). **c**, $\alpha_{xy}/B|_{B\to 0}$ above T_{c0} is plotted against $(T/T_{c0})\ln(T/T_{c0})$ for different values of T_{c0}. Agreement between the data plotted with $T_{c0} = 2.36$ K and Eq. (2) shown by a solid straight line indicates divergence of $\xi_{\rm GL}$ at T_{c0} in the thermal regime ($T > T_{c0}$). **d**, The T dependence of α_{xy}/T at different B above $B_{c2}(0)(= 5.5$ T), which is converted from Fig. 3. Because of the sensitivity limit, α_{xy}/T below $B_{N\to +0}(T)$ is plotted. Solid black lines are guides to the eye. **e**, $(\alpha_{xy}/T)/(\alpha_{xy}/T|_{T\to 0})$ converted from **d** is plotted against T/T^*. Above $B_{N\to +0}(0.1\text{ K})$ $(= 6.1$ T), $\alpha_{xy}/T|_{T\to 0}$ for each B is determined so that the data points of $(\alpha_{xy}/T)/(\alpha_{xy}/T|_{T\to 0})$ for different B collapse on a single curve. The obtained unique curve approximately follows a solid black line given by Eq. (7). A dashed straight line indicates a power-law relation found in the high-T region represented by Eq. (8). **f**, $\alpha_{xy}/T|_{T\to 0}$ above $B_{c2}(0)$ is plotted as a function of $(B'/\widetilde{B}_{c2}(0))\ln(B'/B_{c2}(0))$ for different values of $B_{\rm QCP}$. Agreement between the data plotted with $B_{\rm QCP} = 4.6$ T and Eq. (6) shown by a solid straight line indicates divergence of $\xi_{\rm qf}$ at $B_{\rm QCP}$ in the quantum regime ($B > B_{\rm QCP}$).

2. Correlation length of the amplitude fluctuations in the thermal regime

With increasing B at given T, as explained in the main text, a correlation length of the amplitude fluctuations is reduced from B-independent $\xi_{\rm GL}(T)$ to a magnetic length $l_B(B) = \sqrt{\hbar/2eB}$ when $l_B(B) \leq \xi_{\rm GL}(T)$ above B^*. Then, $\xi_{\rm GL}(T)$ in Eq. (1) is replaced by $l_B(B)$, resulting in $\alpha_{xy}^{\rm fl}/B \propto l_B^2$. Furthermore, for higher $B(\gg B^*)$, the nonlinear correction for this equation is obtained as [10]

$$\frac{\alpha_{xy}^{\rm fl}}{B} \propto \frac{l_B^2}{B} \propto B^{-2}. \tag{3}$$

Consequently, the T and B dependences of $\alpha_{xy}^{\rm fl}/B$ above $T_{\rm c0}$ in arbitrary B follows [10]

$$\frac{\alpha_{xy}^{\rm fl}}{B} = \frac{\alpha_{xy}^{\rm fl}}{B}\Big|_{B\to 0}\frac{1}{1+(B/B^*)^2}. \quad (4)$$

In Fig. S3b, we plot $(\alpha_{xy}/B)/(\alpha_{xy}/B|_{B\to 0})$ against B/B^*, which is converted from the data above $T_{\rm c0}$ in Fig. S3a. All of the data obtained at different T collapse on a unique curve given by Eq. (4) as indicated by a solid black line. This result provides convincing experimental evidence for the existence of the ghost critical field $B^*(T)$, where the expression of $\alpha_{xy}^{\rm fl}$ changes between Eq. (2) and Eq. (3), namely the correlation length switches between $\xi_{\rm GL}(T)$ and $l_B(B)$.

3. Quantum critical behavior in $\alpha_{xy}(T, B)$

The criticality at $B_{\rm QCP}$ is also confirmed by an analysis of $\alpha_{xy}(T)$. As mentioned in the main text, the theoretical value of $\alpha_{xy}^{\rm fl}(T)$ in the low-T region ($T \ll T^*$) for fixed B above $B_{\rm c2}(0)$ is proportional to $\xi_{\rm qf}^2 T$, where $\xi_{\rm qf} = \xi_0/\sqrt{\ln(B/B_{\rm c2}(0))}$ is the correlation length in the quantum regime [5]. The exact theoretical expression of $\alpha_{xy}(T)$ in the quantum regime is given by [8, 9]

$$\frac{\alpha_{xy}^{\rm fl}}{T} = \frac{k_{\rm B}^2}{12\hbar D}\frac{1}{B\ln(B/B_{\rm c2}(0))} \propto \frac{\xi_{\rm qf}^2}{B}. \quad (5)$$

This is a quantum counterpart of Eq. (2). In the present case of $B_{\rm QCP} \neq B_{\rm c2}(0)$, $\xi_{\rm qf}$ should be rewritten as $\xi_{\rm qf} = \xi_0/\sqrt{\ln(B/B_{\rm QCP})}$ and the variable B in Eq. (5) should be replaced by $B(B_{\rm c2}(0)/B_{\rm QCP})$ ($\equiv B' = B(5.5/4.6)$) as deduced from the discussion in the section II. Thus, we obtain

$$\frac{\alpha_{xy}^{\rm fl}}{T} = \frac{k_{\rm B}^2}{12\hbar D}\frac{1}{B'\ln(B'/B_{\rm c2}(0))} \propto \frac{\xi_{\rm qf}^2}{B'}. \quad (6)$$

Therefore, an initial slope of $\alpha_{xy}^{\rm fl}(T)$ in fixed B, i.e., $\alpha_{xy}^{\rm fl}/T|_{T\to 0}$, is a measure of $\xi_{\rm qf}(B)$, which diverges at $B_{\rm QCP}$.

In Fig. S3d, we show the T dependence of α_{xy}/T at different B above $B_{\rm c2}(0)(= 5.5$ T), which is converted from Fig. 3. Because of the sensitivity limit of Nernst signals N, α_{xy}/T below $B_{N\to +0}(T)$ is plotted. α_{xy}/T exhibits a trend to be saturated to a constant value in the low-T region. Note that below $B_{\rm c2}(0)$, α_{xy}/T does not show the saturation behavior but a divergent behavior caused by quantum criticality as discussed in the section IV. In Fig. S3f, we plot $\alpha_{xy}/T|_{T\to 0}$ extracted from Fig. S3d as a function of $(B'/\widetilde{B}_{\rm c2}(0))\ln(B'/B_{\rm c2}(0))$ for different values of $B_{\rm QCP}$ on a log-log scale. A solid straight line corresponds to Eq. (6). We extract the values of $\alpha_{xy}/T|_{T\to 0}$ only in the B region from $B_{\rm c2}(0)$ (= 5.5 T) to $B_{N\to +0}(0.1$ K) (= 6.1 T). As seen in Fig. S3f, the plot of $\alpha_{xy}/T|_{T\to 0}$ against $(B'/\widetilde{B}_{\rm c2}(0))\ln(B'/B_{\rm c2}(0))$ with $B_{\rm QCP} = 4.6$ T is in the best agreement with Eq. (6). The results indicate that criticality at $B_{\rm QCP}$ as well as at $T_{\rm c0}$ is clearly detected by α_{xy} and that $B_{\rm QCP} = 4.6$ T is indeed a quantum critical point that appears associated with the amplitude fluctuations in the quantum regime.

4. Correlation length of the amplitude fluctuations near the quantum regime

In Fig. S3e, similarly to the discussion in the section III-2, we plot $(\alpha_{xy}/T)/(\alpha_{xy}/T|_{T\to 0})$ against T/T^*, which is converted from the data in Fig. S3d. Above $B_{N\to +0}(0.1$ K) (= 6.1 T), the

values of $\alpha_{xy}/T|_{T\to 0}$ for each B are determined so that the data points of $(\alpha_{xy}/T)/(\alpha_{xy}/T|_{T\to 0})$ for different B collapse on a single curve. The obtained unique curve approximately follows

$$\frac{\alpha_{xy}}{T} = \frac{\alpha_{xy}}{T}\Big|_{T\to 0} \frac{1}{1+(T/T^*)^3} \tag{7}$$

as indicated by a solid black line. The observation of the collapse convincingly proves the existence of the ghost temperature $T^*(B)$ as discussed for $B^*(T)$ in the section III-2. From Eq. (7), furthermore, we deduce an empirical expression of $\alpha_{xy}^{\mathrm{fl}}/T$ in the high-T region ($T \gg T^*$) for fixed B as

$$\frac{\alpha_{xy}^{\mathrm{fl}}}{T} \propto T^{-3} \propto \frac{L_\theta^2}{T}, \tag{8}$$

where $L_\theta(T) \sim T^{-1/z}$ is a dephasing length and $z = 1$ is a dynamical critical exponent in charged systems [14]. This expression is interpreted as containing nonlinear correction in the high-T region as seen in Eq. (3) in the high-B region. These results indicate that $T^*(B)$ is a characteristic temperature at which the expression of $\alpha_{xy}^{\mathrm{fl}}$ changes between Eq. (6) and Eq. (8), namely the correlation length of the amplitude fluctuations switches between $\xi_{\mathrm{qf}}(B)$ ($T < T^*$) and $L_\theta(T)$ ($T > T^*$).

IV. Quantum criticality in the anomalous metallic (AM) state

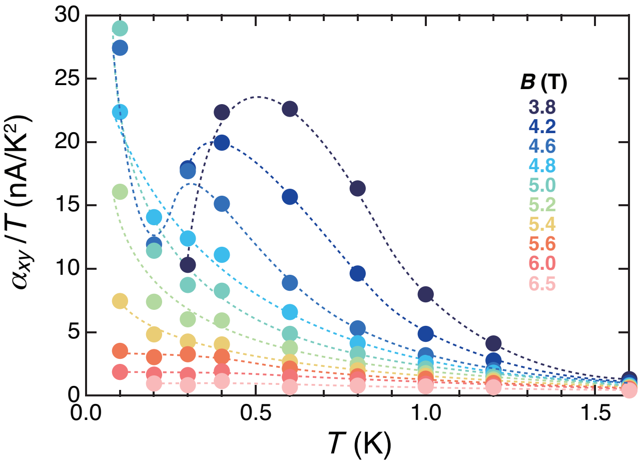

Fig. S4. **Temperature dependence of α_{xy}/T.** α_{xy}/T is plotted as a function of T in the field range across $B_{\mathrm{c2}}(0) = 5.5$ T ($\approx B_{\mathrm{MI}}$). With $T \to 0$, α_{xy}/T saturates to a constant value in $B \geq B_{\mathrm{c2}}(0)$, whereas it shows a trend to diverge in $B < B_{\mathrm{c2}}(0)$, indicative of a quantum critical ground state in the AM state.

Figure S4 shows the T dependence of α_{xy}/T in the field range across $B_{\mathrm{c2}}(0) = 5.5$ T ($\approx B_{\mathrm{MI}}$). In $B \geq B_{\mathrm{c2}}(0)$, α_{xy}/T shows saturation to a constant value as $T \to 0$. This validates the extraction of $\alpha_{xy}/T|_{T\to 0} (\propto \xi_{\mathrm{qf}}(B)^2)$ as discussed in the previous section. On the other hand, in the AM state below $B_{\mathrm{c2}}(0)$, α_{xy}/T shows a trend to diverge as $T \to 0$ and does not converge to a finite value. This indicates a hallmark of a QCP that a temperature derivative of entropy is singular [15–18].

A similar behavior has been found in our previous study of another Mo_xGe_{1-x} thin film [3]. These results strongly suggest that the AM metallic state is a quantum critical ground state.

REFERENCES

[1] Mason, N. and Kapitulnik, A. True superconductivity in a two-dimensional superconducting-insulating system. *Phys. Rev. B* **64**, 060504(R) (2001).

[2] Sato, H. and Okuma, S. Absence of mode-locking resonance for driven vortices in a thin amorphous Mo_xGe_{1-x} film. *J. Phys.: Conf. Ser.* **400**, 022103 (2012).

[3] Ienaga, K., Hayashi, T., Tamoto, Y., Kaneko, S., and Okuma, S. Quantum Criticality inside the Anomalous Metallic State of a Disordered Superconducting Thin Film. *Phys. Rev. Lett.* **125**, 257001 (2020).

[4] Fisher, D. S., Fisher, M. P. A., and Huse, D. A. Thermal fluctuations, quenched disorder, phase transitions, and transport in type-II superconductors. *Phys. Rev. B* **43**, 130-159 (1991).

[5] Varlamov, A. A., Galda, A., and Glatz, A. Fluctuation spectroscopy: From Rayleigh-Jeans waves to Abrikosov vortex clusters. *Rev. Mod. Phys.* **90**, 015009 (2018).

[6] Glatz, A., Pourret, A., and Varlamov, A. A. Analysis of the ghost and mirror fields in the Nernst signal induced by superconducting fluctuations. *Phys. Rev. B* **102**, 174507 (2020).

[7] Ussishkin, I., Sondhi, S. L., and Huse, D. A. Gaussian superconducting fluctuations, thermal transport, and the Nernst effect. *Phys. Rev. Lett.* **89**, 287001 (2002).

[8] Serbyn, M. N., Skvortsov, M. A., Varlamov, A. A., and Galitski, V. Giant Nernst effect due to fluctuating Cooper pairs in superconductors. *Phys. Rev. Lett.* **102**, 067001 (2009).

[9] Michaeli, K. and Finkel'stein, A. M. Quantum kinetic approach to the calculation of the Nernst effect. *Phys. Rev. B* **80**, 214516 (2009).

[10] Pourret, A., Aubin, H., Lesueur, J., Marrache-Kikuchi, C. A., Bergé, L., Dumoulin, L., and Behnia, K. Length scale for the superconducting Nernst signal above T_c in $Nb_{0.15}Si_{0.85}$. *Phys. Rev. B* **76**, 214504 (2007).

[11] Pourret, A., Spathis, P., Aubin, H., and Behnia, K. Nernst effect as a probe of superconducting fluctuations in disordered thin films. *New J. Phys.* **11**, 055071 (2009).

[12] Pourret, A., Aubin, H., Lesueur, J., Marrache-Kikuchi, C. A., Berge, L., Dumoulin, L., and Behnia, K. Observation of the Nernst signal generated by fluctuating Cooper pairs. *Nature Phys.* **2**, 683-686 (2006).

[13] Blatter, G., Feigel'man, M. V., Geshkenbein, V. B., Larkin, A. I., and Vinokur, V. M. Vortices in high-temperature superconductors. *Rev. Mod. Phys.* **66**, 1125-1388 (1994).

[14] Sondhi, S. L., Girvin, S. M., Carini, J., and Shahar, D. Continuous quantum phase transitions. *Rev. Mod. Phys.* **69**, 315-333 (1997).

[15] Vojta, M. Quantum phase transitions. *Rep. Prog. Phys.* **66**, 2069-2110 (2003).

[16] Löhneysen, H. v., Rosch, A., Vojta, M., and Wölfle, P. Fermi-liquid instabilities at magnetic quantum phase transitions. *Rev. Mod. Phys.* **79**, 1015-1075 (2007).

[17] Izawa, K., Behnia, K., Matsuda, Y., Shishido, H., Settai, R., Onuki, Y., and Flouquet, J. Thermoelectric response near a quantum critical point: the case of $CeCoIn_5$. *Phys. Rev. Lett.* **99**, 147005 (2007).

[18] Machida, Y., Tomokuni, K., Ogura, C., Izawa, K., Kuga, K., Nakatsuji, S., Lapertot, G., Knebel, G., Brison, J.-P., and Flouquet, J. Thermoelectric response near a quantum critical point of β-$YbAlB_4$ and $YbRh_2Si_2$: a comparative study. *Phys. Rev. Lett.* **109**, 156405 (2012).
